# TriMEDB: A database to integrate transcribed markers and facilitate genetic studies of the tribe Triticeae

**DOI:** 10.1186/1471-2229-8-72

**Published:** 2008-06-30

**Authors:** Keiichi Mochida, Daisuke Saisho, Takuhiro Yoshida, Tetsuya Sakurai, Kazuo Shinozaki

**Affiliations:** 1 Plant Science Center, RIKEN, Yokohama 230-0045, Japan; 2 Research Institute for Bioresources, Okayama University, Kurashiki 710-0046, Japan

## Abstract

**Background:**

The recent rapid accumulation of sequence resources of various crop species ensures an improvement in the genetics approach, including quantitative trait loci (QTL) analysis as well as the holistic population analysis and association mapping of natural variations. Because the tribe Triticeae includes important cereals such as wheat and barley, integration of information on the genetic markers in these crops should effectively accelerate map-based genetic studies on Triticeae species and lead to the discovery of key loci involved in plant productivity, which can contribute to sustainable food production. Therefore, informatics applications and a semantic knowledgebase of genome-wide markers are required for the integration of information on and further development of genetic markers in wheat and barley in order to advance conventional marker-assisted genetic analyses and population genomics of Triticeae species.

**Description:**

The Triticeae mapped expressed sequence tag (EST) database (TriMEDB) provides information, along with various annotations, regarding mapped cDNA markers that are related to barley and their homologues in wheat. The current version of TriMEDB provides map-location data for barley and wheat ESTs that were retrieved from 3 published barley linkage maps (the barley single nucleotide polymorphism database of the Scottish Crop Research Institute, the barley transcript map of Leibniz Institute of Plant Genetics and Crop Plant Research, and HarvEST barley ver. 1.63) and 1 diploid wheat map. These data were imported to CMap to allow the visualization of the map positions of the ESTs and interrelationships of these ESTs with public gene models and representative cDNA sequences. The retrieved cDNA sequences corresponding to each EST marker were assigned to the rice genome to predict an exon-intron structure. Furthermore, to generate a unique set of EST markers in Triticeae plants among the public domain, 3472 markers were assembled to form 2737 unique marker groups as contigs. These contigs were applied for pairwise comparison among linkage maps obtained from different EST map resources.

**Conclusion:**

TriMEDB provides information regarding transcribed genetic markers and functions as a semantic knowledgebase offering an informatics facility for the acceleration of QTL analysis and for population genetics studies of Triticeae.

## Background

Accumulation and saturation of available genetic markers directly contribute to advances in marker-assisted genetic studies with a wide range of applications. Genetic markers designed to extensively cover a genome permit not only the detection and identification of individual genes associated with complex traits by quantitative trait loci (QTL) analysis but also the exploration of genetic diversity and population structure with regard to natural variations [1-3]. The recent rapid accumulation of sequence resources of various crop species ensures an improvement in the genetics approach in combination with comparative genomics [4]. The increasing availability of crop genome resources has greatly facilitated the elucidation of crop evolution; this elucidation involves the discovery of key loci, thereby contributing to genetic-based domestication and improvement [5].

The tribe Triticeae includes important crops such as wheat (*Triticum aestivum *L.) and barley (*Hordeum vulgare *L.). It is necessary to accumulate and saturate the available markers and to design a core marker set for efficient map-based research in order to discover the key loci associated with phenotypic changes that occur in different varieties of these crops and are involved in productivity. These efforts will contribute to sustainable food production through the application of molecular breeding. Expressed sequence tags (ESTs) of common wheat and barley have been collected on a large scale in order to establish a comprehensive sequence resource for gene discovery and a reliable database of gene expression [6,7]. The number of ESTs and non-redundant sequences of these crops have dramatically increased in recent years. On March 1, 2008, the UniGene database of NCBI contained 41,227 and 23,078 representative sequences of clustered ESTs of wheat and barley, respectively. These comprehensive EST collections have a potential application in the development of genome-wide genetic markers. Thus far, different barley maps have been constructed using EST-derived markers [8-10]; consequently, the potential availability of holistic genetic markers in the Triticeae genome has increased. The number of genetic markers designed from ESTs is more than 3000; this number is published along with the chromosome location of these ESTs in the barley genome. Recently, a genetic map of diploid wheat (*Triticum monococcum *L.) was constructed using transferable markers derived from barley ESTs [11]. This approach clearly demonstrates that EST markers derived from barley EST data can be directly used as genetic markers for mapping the wheat genome. Therefore, interrelating and integrating the EST markers in barley with their homologues in wheat is a valid and reliable expedient to enhance and accumulate potential EST markers in both barley and wheat, because the homoeologous linkage groups between diploid wheat and barley are remarkably conserved [11,12].

Recently, a genetic approach that uses multiple mapping populations for comparison and integration of QTLs across populations has been reported; this approach is quite effective for the extensive investigation of the genetic architecture of genome-wide complex traits. For instance, seed dormancy QTLs in barley have been detected using multiple mapping populations composed of 7 recombinant inbred (RI) and 1 doubled haploid (DH) populations, in order to integrate and extend the use of previously known QTLs and phenotypes for the evaluation of diverse germplasms. Consequently, conserved QTLs among populations and coincident QTLs have been identified using consensus marker intervals [13]. More recently, nested association mapping (NAM) strategy, which simultaneously exploits the advantages of both linkage analysis and association mapping, has been implemented in maize as a new complex trait dissection strategy; this strategy involves the genotyping of common-parents-specific (CPS) markers for RI line populations that have been produced by a diverse set of parents and a common parental line [14]. For genetic approaches of a genome-wide nature, unification and integration of the available cDNA markers to generate a common marker set across populations would facilitate high-throughput genotyping for multiple populations and/or natural variations.

Thus, the integration of the available cDNA markers into a semantic knowledgebase that can provide various annotations and generate a unified set of identical markers would definitely be useful for improving the genome-wide map-based approaches. Herein, we report the Triticeae mapped EST database (TriMEDB) – a database providing information on the mapped cDNA markers in barley, along with various annotations, and their homologues in wheat. The current version of TriMEDB contains 2737 unique cDNAs mapped onto linkage maps, and the results of queried data are displayed on the web interface.

## Construction and content

### EST marker source

The current version of TriMEDB provides information regarding barley and wheat ESTs and their map location data retrieved from 3 published barley linkage maps and 1 diploid wheat map. This information can be imported to CMap [15] and used to visualize the map positions of the ESTs. The EST sequences, polymerase chain reaction (PCR) primers, and map positions of 1052 EST-based barley markers derived from the Leibniz Institute of Plant Genetics and Crop Plant Research (IPK) were retrieved from the National Center for Biotechnology Information (NCBI) GenBank by using the accession numbers available therein. The barley SNP database of Scottish Crop Research Institute (SCRI) for 332 markers and the data for CMap were retrieved from the SCRI website. The sequence and map positions of the contigs for 1848 markers were also retrieved using HarvEST barley ver. 1.63. A total of 240 PCR markers of diploid wheat were obtained from the literature of Hori *et al*. (2007). These PCR markers were used to detect the corresponding sequences in the wheat and barley EST sets in dbEST (GenBank EST release #163.0). The longest ESTs found by this search for each PCR primer were analysed further by studying the cDNA sequence corresponding to each primer pair.

### Assignment of EST markers to gene models of public sequence resources

Nucleotide sequences of the mapped EST markers were obtained from each cDNA database that provides representative sequences of clustered or assembled cDNA. For this purpose, PCR primer sequences and amplified marker sequences were searched for NCBI UniGene [16], TIGR Gene Index [17], HarvEST [10], and the Plant Genome Database (PlantGDB) [18] for barley by matching the sequence identity of the PCR primers or by performing a similarity search using BLASTN at a threshold e value of <1e × 10^-200^. Marker sequences that were homologous between barley and wheat were identified using BLASTN similarity searches against each sequence resource with a threshold e value of <1e × 10^-130^.

### Unification of EST markers

To generate a unique EST marker set for the tribe Triticeae, 3472 ESTs derived from 4 maps were assembled using CAP3 [19] with default parameter settings; these ESTs were grouped into 2737 unique marker groups as contigs. These contigs were used as virtual markers to compare the linkage maps of homoeologous chromosomes.

### Assignment of markers onto the rice genome

To identify the homologous counterparts of the rice gene for each barley EST marker and to predict an exon-intron structure based on the rice homologous sequences, a homology search was performed using BLASTN with a threshold e value of <1e × 10^-20 ^against the rice genome sequence of The International Rice Genome Sequencing Project (IRGSP) ver. 4.0 obtained from the Rice Annotation Project Database (RAP-DB) [20]. This database was used to approximately locate and extract the homologous regions, and the homologous pairs between the cDNA sequences of the markers and a rice genome fragment that covers 5-kb sequence of both the flanked homologous regions obtained via BLASTN search were aligned using SIM4 [21] with default parameter settings. The predicted exon-intron structure data was applied to the in-house database along with the rice genome annotation dataset which was obtained from RAP-DB by using Generic Genome Browser [22].

### Database and web interface

The database is implemented in MySQL and Perl CGI scripts and the web interface runs on the Apache Web server. CMap is implemented to visualize the linkage maps. Generic Genome Browser is implemented to display the exon-intron structure of the cDNAs of each marker and to compare the ESTs with the rice homologous sequences and the annotated rice genome obtained from RAP-DB.

## Utility

### Marker search

**Figure 1:**
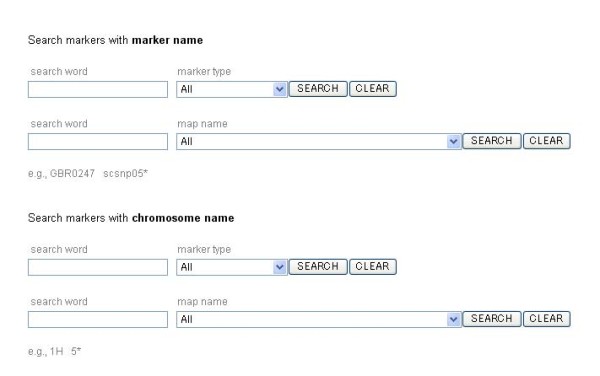
TriMEDB web interface used for marker search. A user can search markers by entering a marker name along with the marker type or the name of a genetic map listed in the drop-down menu, and then filter the search result by entering the chromosome name in order to retrieve the markers located on each chromosome. All the search text fields accept the wild card '*', which is used to indicate an arbitrary word as the query.

**Figure 2:**
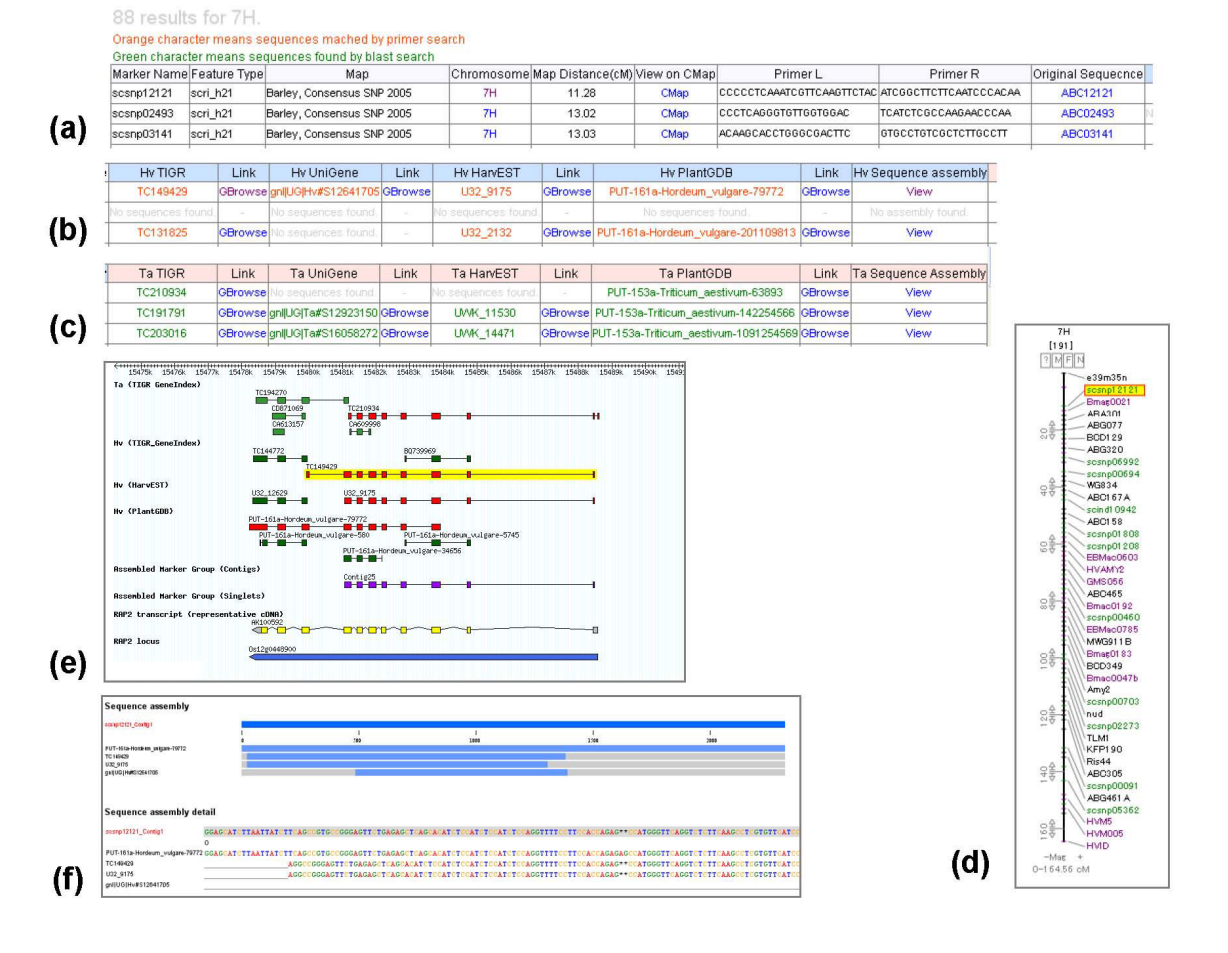
The result of an example marker search performed using '7H' as the query. The spreadsheet of the result page of the marker search comprises tables displaying general information obtained from each marker resource (a). Identifiers of the representative sequences of barley (b) and those for wheat (c) on TIGR GI, NCBI UniGene, HarvEST, and PlantGDB are listed. The hyperlink of the chromosome name links to the linkage map on CMap (d). The Gbrowse page is used to visualize the predicted exon-intron structure of the cDNA sequences of each marker along with the rice gene annotations released by RAP-DB (e). The contig assembly of cDNA sequences that correspond to each marker is displayed (f).

A user can search the EST markers in barley and diploid wheat by using 2 different queries: a marker name and a chromosome name (Fig. 1). Each of the 2 queries can be filtered according to the marker type, such as SNP and cleaved amplified polymorphic sequence (CAPS), and the name of the linkage map. A wild card '*' is acceptable in the search text fields, i.e. the field provided for retrieving all markers located on a certain chromosome. The results for the searched marker are displayed in the form of a spreadsheet consisting of 3 major divisions, as shown in Fig. 2a, 2b, and 2c. The left division consists of 9 columns and displays general information such as marker name, marker type, original mapping population, chromosome location and map position, and PCR primers and/or original sequence retrieved from each marker resource (Fig. 2a). The other 2 divisions display the representative sequences of cDNAs derived from the following 4 individual sequence resources: TIGR Gene Index, NCBI UniGene, HarvEST, and PlantGDB. These representative sequences correspond to each marker that has been identified by the primer sequence search and/or sequence similarity search. Of the other 2 divisions, one provides barley-related information while the other provides wheat-related information. Each of these 2 divisions has 3 types of hyperlinks (Fig. 2b, 2c). The hyperlink of the chromosome name links to linkage maps that can be visualized on CMap (Fig. 2d). The hyperlinks of the sequence identifiers link to a page showing individual FASTA-formatted sequences of each sequence resource and homology search results of BLASTN that have been obtained using the original marker sequence as the query. The hyperlink of 'Gbrowse' links to a local generic genome browser that provides the annotated rice genome released from RAP-DB and displays the exon-intron structure of the cDNAs of the markers that has been derived on the basis of the homologous sequences of the rice genome (Fig. 2e). The hyperlinks of 'View' in the sequence assembly columns link to a page showing the contig assembly containing corresponding sequences derived from the sequence resources and provides contig assemblies for the longest cDNA sequence currently available (Fig. 2f).

### Assembled marker search

**Figure 3:**
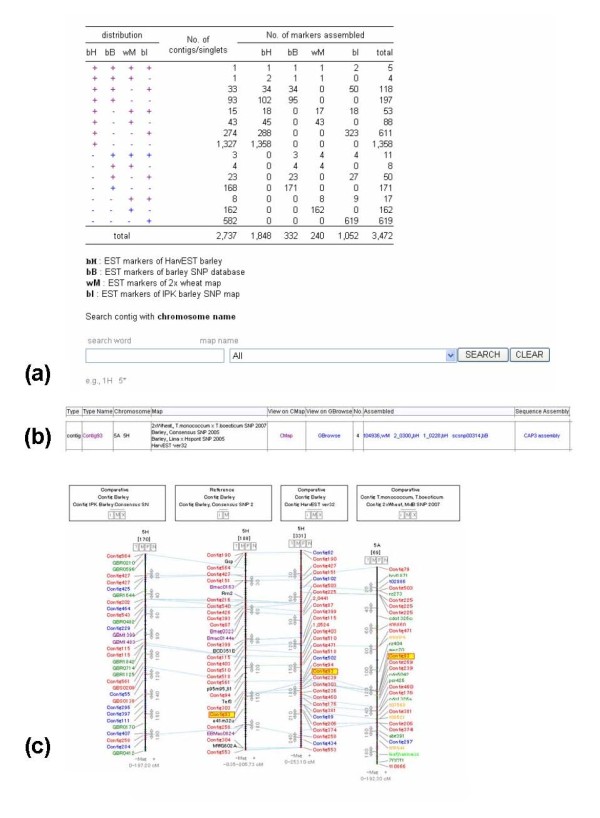
Web interface for representing unified markers. A web page showing the table that presents the distribution status of identical markers derived from 4 individual marker resources of barley and diploid wheat. A user can access the unified marker list by clicking each link in the distribution pattern and performing a search by entering a chromosome name along with the linkage map name (a). The unified markers are listed as contigs along with their members and are hyperlinked to CMap, where the linkage maps from different resources can be compared. As an example, Contig93 which is common between 2 barley maps and a diploid wheat map is hyperlinked to CMap, Gbrowse, assembled marker information, and CAP3 assembly (b). Contig assembly of identical markers is used for virtual markers to compare linkage maps among map resources. A comparison of the linkage maps of homoeologous group 5 among 3 different barley maps and a diploid wheat map performed using assembled marker groups shared among them is depicted (c).

The assembled marker search page provides an interface to retrieve 2737 unique marker groups derived from the CAP3 assembly for a total of 3472 markers. As shown in Fig. 3a, the web interface of this page describes the number of markers and the distribution among 4 individual EST marker resources. Each hyperlink showing individual distribution of markers links to a page representing the contig list along with the contig identification tag and a hyperlink to CMap, list of assembled markers, and a link to the contig assembly (Fig. 3b). Users can search the assembled markers by using the chromosome name and the linkage map name in order to retrieve a unified marker set located on a target chromosome (Fig. 3a). The linkage map linked to this page provides comparative illustration of multiple linkage maps that have been obtained using these virtual markers unified into the same contigs; this map is useful for identifying not only the markers that are common among different linkage maps but also possible candidates that can be transferred from other linkage maps (Fig. 3c).

## Discussion and Conclusion

Accumulation of information pertaining to the genetic markers in barley and wheat and integration of various annotations related to these markers into a semantic knowledgebase have been effective in facilitating the map-based approach for Triticeae genomics. Our assembly that unifies the mapped EST markers currently available for barley and wheat has already been used to map more than 2700 cDNAs to homoeologous chromosomes of barley and wheat. These unique markers would be considered initial candidates with regard to polymorphisms that can be used for generating linkage maps by using a novel mapping populations that may in turn be used for QTL analysis. These unique markers would be shared by barley and wheat. Because the predicted exon-intron structures and the information on the rice genome should also be useful in designing PCR primers to amplify suitable introns for polymorphism discovery, they should also benefit the effective throughput genome mapping of Triticeae [23].

Genetic markers are provided by several database resources, which have a web interface to allow the users to browse genetic linkage maps. GrainGenes is a popular site for Triticeae genomics; it also provides genetic markers and linkage map data on wheat, barley, rye, and oat [24]. Gramene is a database for plant comparative genomics; it provides genetic maps of various plant species [25]. TriMEDB focuses on mapped ESTs; compared to these previously released databases, TriMEDB allows a greater utility of genetically mapped ESTs in Triticeae because of its 3 specific features. (1) Genetically mapped EST markers in barley and diploid wheat have been assigned to clustered EST sequence databases, which are public databases of wheat and barley, namely, UniGene, HarvEST, TIGR Gene Index, and Plant GDB. With this database, users can find homologous sequences of the markers in both the species, as shown in Fig. 2b and 2c. (2) Furthermore, the sequences of clustered ESTs have been mapped on the rice genome on the basis of sequence similarity in order to view the predicted exon-intron structure and sinteny between Triticeae and rice. (3) In this database, EST markers have been clustered and assembled into contig groups to unify the EST markers derived from various resources. Therefore, a user can directly compare genetic maps among barley as well as between barley and diploid wheat. We believe that these features of TriMEDB would be beneficial for the improvement in cereal and grass genomics.

Supplemental Figure. S1 in Additional File 1 illustrates an example use of TriMEDB as a semantic knowledgebase of Triticeae EST markers with respect to the queries encountered in map-based studies of grass genomics. TriMEDB can be useful in queries such as those involving the search of markers to perform genome-wide genotyping for linkage map constructions or linkage disequilibrium (LD) mapping. Unified contig markers could be more applicable in the case of QTL comparison among multiple mapping populations and cross-Triticeae species. As shown in Fig. 3, barley EST markers derived from different genetic maps were assembled into identical marker groups and imported to CMap for comparison. Consequently, TriMEDB may function as an informatics gateway to perform cross-Triticeae genomics [9]. Moreover, it may be useful for the integrative analysis of genetic knowledge among the various varieties of barleys as well as for comparison of conserved QTLs on homoeologous chromosomes between barley and wheat.

Furthermore, the derived marker groups may be applicable as a set of core markers for high-throughput genotyping of natural variation in order to display the population structure of Triticeae plants. Domestication and adaptation have lead to the expansion of the area of cultivation of wheat and barley [26]. TriMEDB would definitely contribute to Triticeae genomics because it would help in discovering the key loci associated with adaptation to various environments and may contribute to the discovery of phenotypic variations in domesticated varieties. TriMEDB may also be applicable to EST marker accumulation onto the target chromosomal regions of detected QTLs by search more markers which should be allocated on intervals of commonly located markers. A search for a candidate gene on the basis of Triticeae/rice genome colinearity might be useful for browsing rice genome annotation displayed on Gbrowse. TriMEDB can incorporate any mapped EST data as semantic knowledge if the data includes the marker name and any of the following sequence identifiers: accession ID of a public database, sequences of PCR primers, or marker sequence [See supplemental Figure S2 in Additional File 1]. Therefore, it is possible to update and further accumulate mapped EST information onto TriMEDB. These aspects of TriMEDB as a new repository of functional genetic markers would allow us to promote robust QTL analysis using multiple segregating populations, population analysis and genome wide association mapping, and those combined approach in Triticeae species. This novel database may develop as a platform for projects involving marker saturation, narrowing down of QTL regions and those cloning, and comparative grass genomics.

Therefore, TriMEDB is particularly useful for both conventional map-based analyses, such as QTL analysis and association mapping, and evolutionary population genomics studies that will facilitate the molecular breeding of Triticeae plants.

## Availability and requirements

Project name: TriMEDB: Triticeae Mapped EST Database

Project home page:

Operating system: Platform independent

Programming language: Perl

Other requirements: None

License: None required

Any restrictions to use by non-academicians: None

## Authors' contributions

KM designed the project, performed data analysis, assisted in the designing of the database, and drafted the manuscript. DS also designed the project and analysed the published genetic maps. TY performed data analysis and assisted in the designing of the web interface and the database. TS performed database system administration. KS served as the principal investigator of the project. All authors have contributed to the writing of the manuscript and have read and approved the final submitted version.

## Supplementary Material

Additional file 1 Supplemental Figure S1 is a schematic presentation of the map-based analyses and the use of TriMEDB. Supplemental Figure S2 is a schematic presentation of the database structure of TriMEDB. Click here for file
