# Supplementary material for: TriMEDB: A database to integrate transcribed markers and facilitate genetic studies of the tribe Triticeae
Source: BMC Plant Biol. 2008 Jun 30;8:72. doi: 10.1186/1471-2229-8-72 (PMC2474609; doi:10.1186/1471-2229-8-72)
Supplement: Additional file 1 — Supplemental Figure S1 is a schematic presentation of the map-based analyses and the use of TriMEDB. Supplemental Figure S2 is a schematic presentation of the database structure of TriMEDB. [file 1471-2229-8-72-S1.pdf]

Supplemental Material

Supplemental Figure S1. Schematic presentation of the map-based analyses and the use of TriMEDB.

Supplemental Figure S2. Schematic presentation of the database structure of TriMEDB.

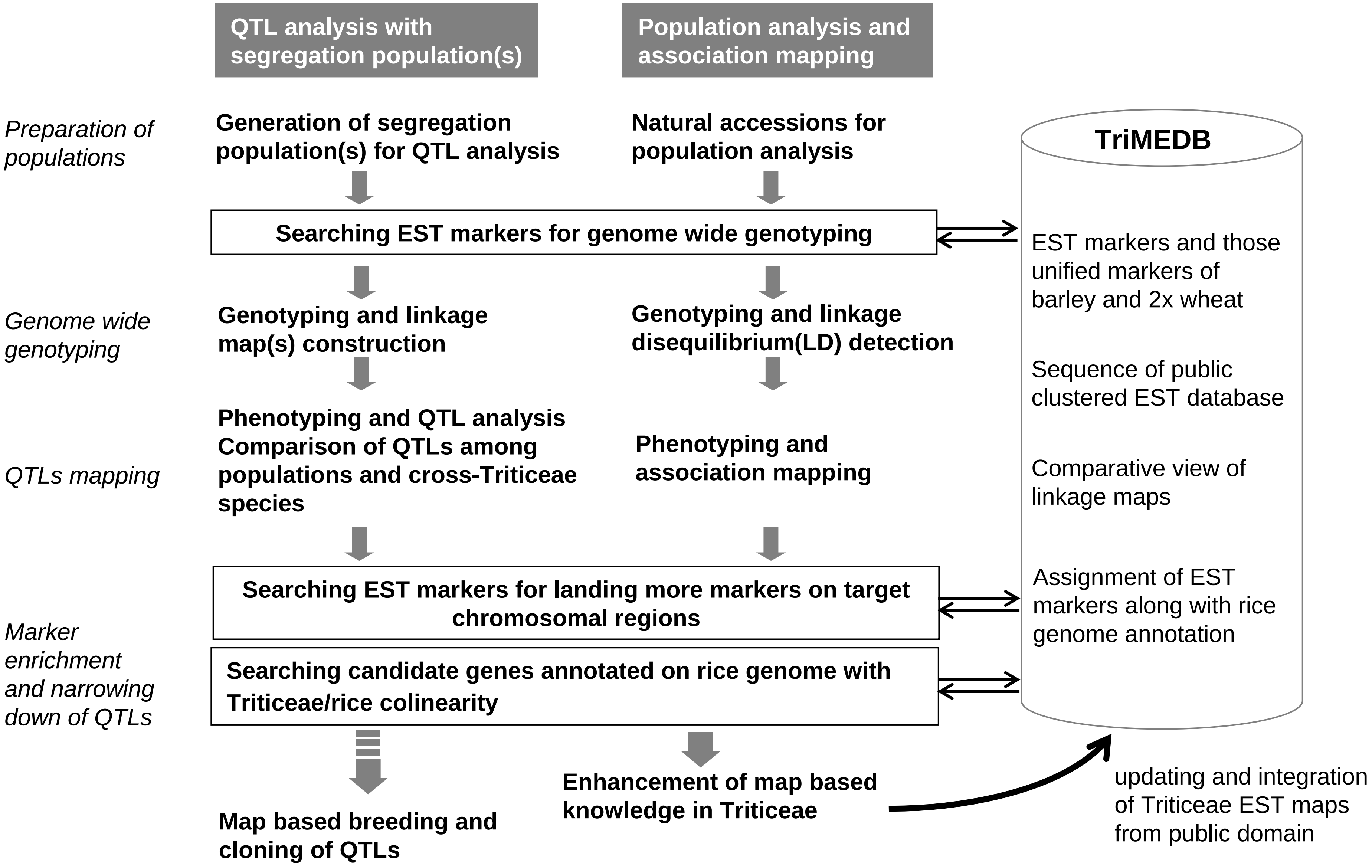

Supplemental Figure S1:

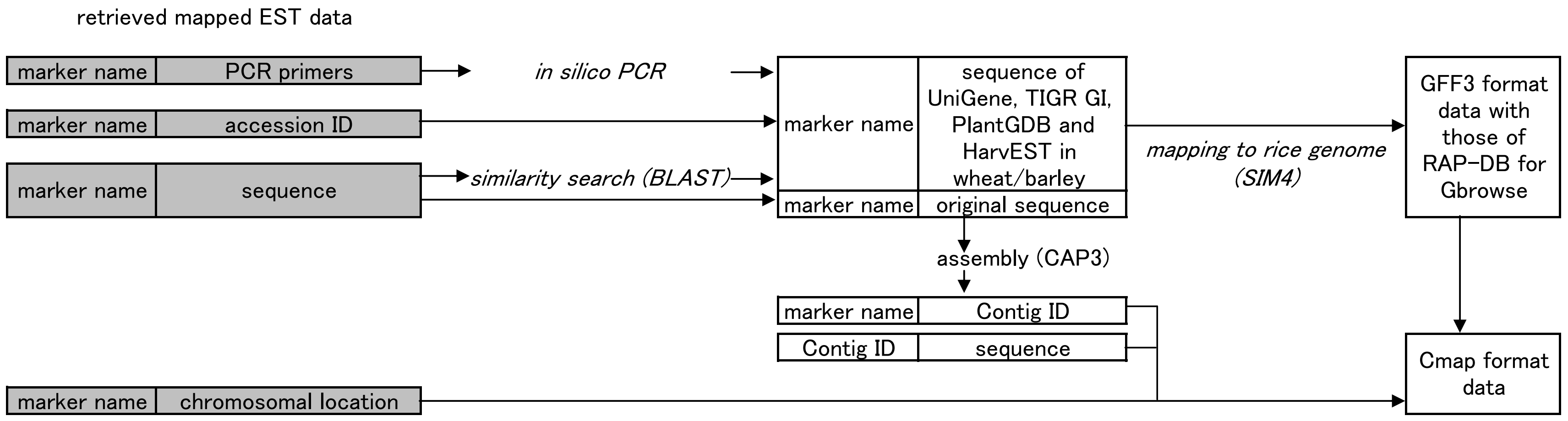

Supplemental Figure S2:
